# Supplementary material for: Subsurface hydrocarbon degradation strategies in low- and high-sulfate coal seam communities identified with activity-based metagenomics
Source: NPJ Biofilms Microbiomes. 2022 Feb 17;8:7. doi: 10.1038/s41522-022-00267-2 (PMC8854433; doi:10.1038/s41522-022-00267-2)
Supplement: Supplementary file 1 — Supplemental Information File [file 41522_2022_267_MOESM1_ESM.pdf]

Subsurface Hydrocarbon Degradation Strategies in Low- and High-Sulfate Coal Seam Communities Identified with Activity-Based Metagenomics

Authors: Hannah Schweitzer[1,2,†§**], Heidi Smith[1,2,§ **], Elliott P. Barnhart[1,3], Luke McKay[1,4], Robin Gerlach[1,5,6], Alfred B. Cunningham[1,5,7], Rex R. Malmstrom[8], Danielle Goudeau[8], and Matthew W. Fields[1,2,5]**

Affiliations:

[1]Center for Biofilm Engineering, Montana State University, Bozeman, MT 59717, USA

[2]Department of Microbiology & Cell Biology, Montana State University, Bozeman, MT 59717, USA

[3]US Geological Survey, Wyoming-Montana Water Science Center, Helena, MT 59601,USA

[4]Department of Land Resources and Environmental Sciences, Montana State University, Bozeman, MT 59717, USA

[5]Energy Research Institute, Montana State University, Bozeman, MT 59717, USA

[6]Department of Biological and Chemical Engineering, Montana State University, Bozeman, MT 59717, USA

[7]Department of Civil Engineering, Montana State University, Bozeman, MT 59717, USA

[8]DOE Joint Genome Institute, Berkeley, CA 94720, USA

[§]Indicates both authors contributed equally to this work

[†]Now at UiT - The Arctic University of Norway, 9019 Tromsø, Norway

[**]Corresponding authors

H.D. Schweitzer, Post Doctoral Researcher

UiT - The Arctic University of Norway

The Norweigian College of Fishery Science

Muninbakken 21

9019 Tromsø, Norway

hannah.schweitzer@uit.no

H.J. Smith, Assistant Research Professor

Montana State University

Center for Biofilm Engineering

366 EPS Building

Bozeman, MT 59717, USA

Heidi.smith@montana.edu

M.W. Fields, Professor
Montana State University
Center for Biofilm Engineering
366 EPS Building
Bozeman, MT 59717, USA
Matthew.fields@montana.edu

Supplementary Information Contents:

Supplementary Figures 1-3

Supplementary Tables 1-5 and Supplementary Data 1-3

Supplementary **Figures:**

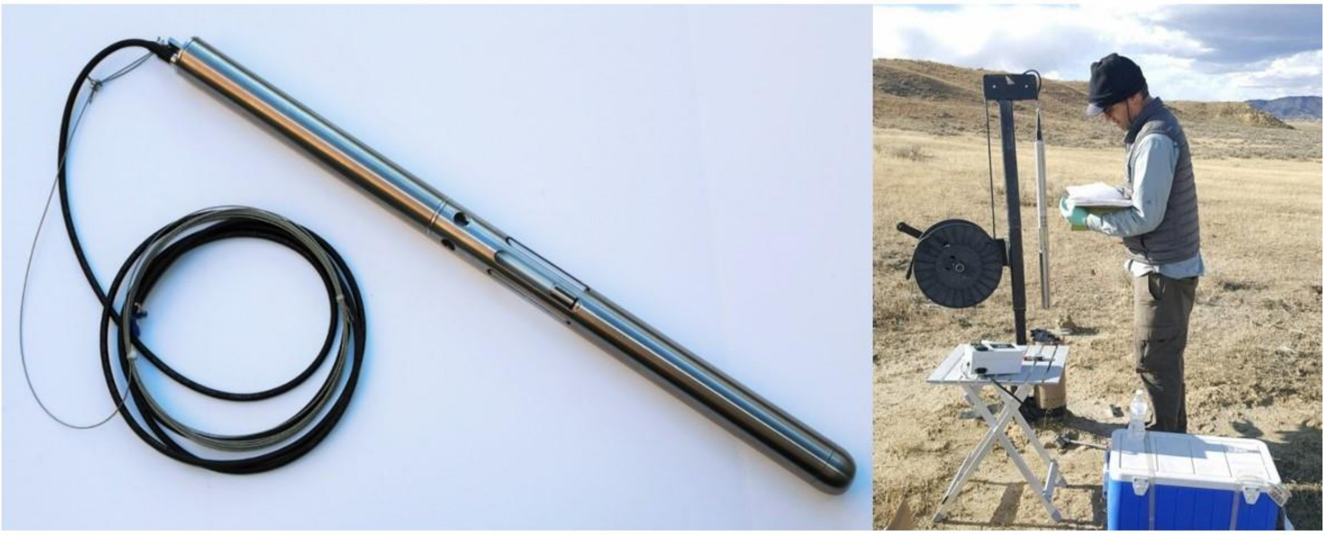

Supplementary Figure 1.) Photographs of the Subsurface Environmental Sampler (SES) (image on right, courtesy of Jesse Barnhart) used to obtain coal associated communities. The authors affirm that the individual imaged in the right provided informed consent for publication of the image.

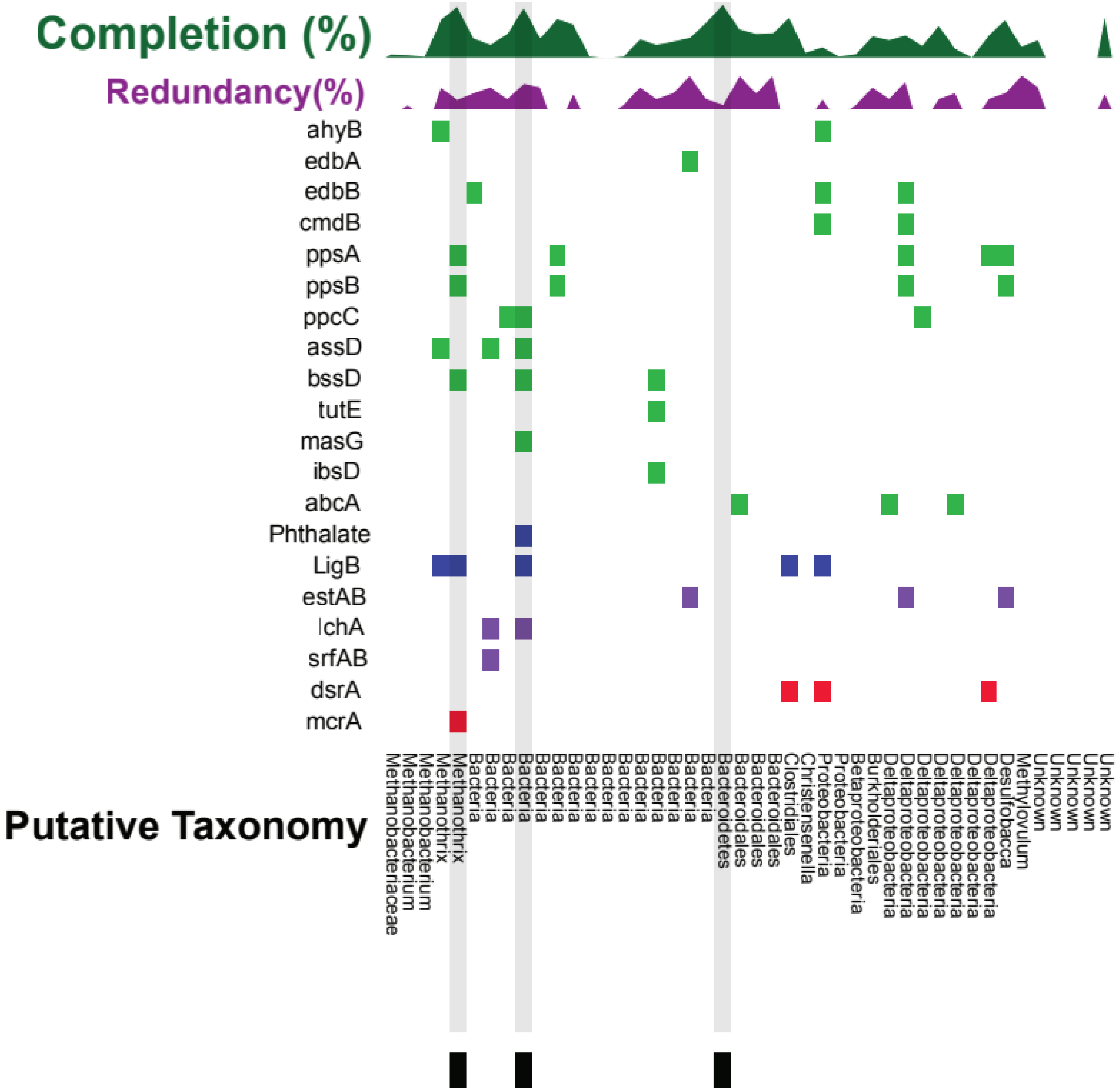

Supplementary Figure 2.) Evaluation of SYTO-Total metagenome assembled genomes (MAGs) and the presence or absence of anaerobic (green) and aerobic hydrocarbon degrading (blue), biosurfactant (purple) and sulfate reduction/methanogenesis (red) genes for each corresponding MAG. The AnHyDeg and AromaDeg databases were used to determine the different hydrocarbon degradation genes used for comparison. The percent completion (green line graph out of 100%) and redundancy (purple line graph out of 10%) were compared for each MAG. MAGs with putative taxonomy Methanothrix, Bacteria and Bacteriodetes were all greater than 92% complete and contain an average nucleotide identity that is >99% similar to their matching BONCAT MAGs which is represented by the symbol (■).

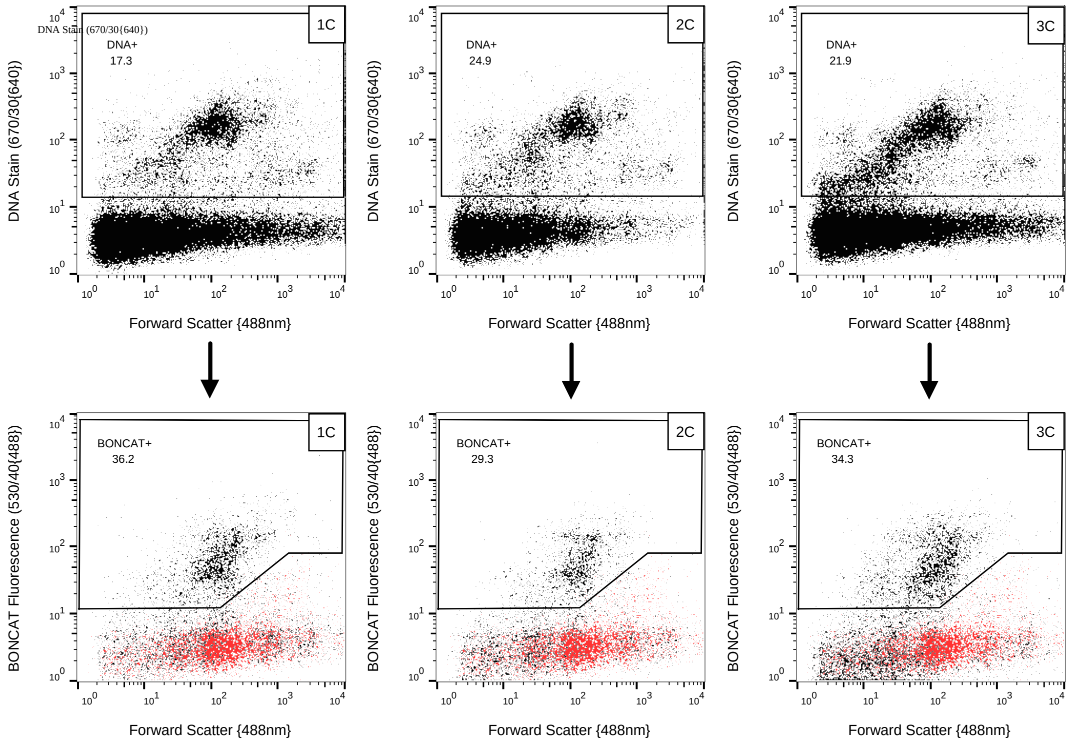

Supplementary Figure 3.) Gate drawing accomplished in two steps for the biological replicates of FG-11 BONCAT positive samples. The top panel 1A, 2A, and 3A is gated to separate total cells from background particles and gates indicated in black are based on a DNA stain (Syto59, ex: 640nm em: 655-685nm). Following the initial gating, SYTO+ cells were analyzed further for BONCAT fluorescence with the FAM (Picolyl dye (Ex: 488nm/Em: 530nm) (bottom panel). Gate for 1B, 2B, and 3B. Gating of the BONCAT+ populations was achieved by comparing each replicate to both HPG negative and water controls (indicated in red overlaid in each graph). The number in the top left of each of the bottom panel graphs indicates the percentage of BONCAT+ cells in comparison to total cells (SYTO+) for each replicate.

Supplementary Tables/Data:

***Uploaded as a data file with table explanations provided below**

Supplementary Table 1.) List of environmental metagenome assembled genomes (MAGs) from high sulfate and low sulfate coal seams from the Powder River Basin and the corresponding sequencing and analysis parameters.

Supplementary Table 2.) List of BONCAT+ metagenome assembled genomes (MAGs) and the corresponding sequencing and analysis parameters. The corresponding genes present in each MAG are listed under their corresponding database.

Supplementary Table 3.) List of SYTO-Total metagenome assembled genomes (MAGs) and there corresponding sequencing and analysis parameters. The corresponding genes that are present in each MAG are listed under their corresponding database.

Supplementary Table 4.) Complete list of the four databases and all the genes compared in this study. The function of each gene and the coverage of each gene in each sample is listed and the presence of gene in the BONCAT+ sample is indicated. The genes further analyzed are marked as genes of interest.

Supplementary Table 5.) Formation water and dissolved gas chemical and isotopic composition. Proximate/ultimate analysis percentages and heating values of the coal. All data reported in this table was modified and described in more depth in Barnhart et al. 2016.

Supplementary Data 1.) List of functional genes from the high- and low-sulfate coal seams determined with the Kyoto Encyclopedia of Genes and Genomes (KEGG) gene database. The BONCAT+ sample is the translationally active community from the low-sulfate (FG-L) coal seam. NaN means there was no gene abundance for that particular metabolic pathway identified in that sample.

Supplementary Data 2.) The gene abundances (calculated with RPKM) of individual genes of interest involved in hydrocarbon degradation for the shotgun environmental MAGs from the high-sulfate coal seam (N-H) and two low-sulfate coal seams (T-L and FG-L). All MAGs that were below 80% completion were grouped together as <80% Completion.

Supplementary Data 3.) Average Nucleotide Identity comparison of all shotgun, BONCAT+ and SYTO-Total metagenomes.
