# Supplementary material for: Subsurface hydrocarbon degradation strategies in low- and high-sulfate coal seam communities identified with activity-based metagenomics
Source: NPJ Biofilms Microbiomes. 2022 Feb 17;8:7. doi: 10.1038/s41522-022-00267-2 (PMC8854433; doi:10.1038/s41522-022-00267-2)
Supplement: Supplementary file 10 — Reporting Summary [file 41522_2022_267_MOESM10_ESM.pdf]

nature portfolio

Corresponding author(s): Hannah Schweitzer, Heidi Smith, Matthew Fields

Last updated by author(s): Nov 29, 2021

Reporting Summary

Nature Portfolio wishes to improve the reproducibility of the work that we publish. This form provides structure for consistency and transparency in reporting. For further information on Nature Portfolio policies, see our Editorial Policies and the Editorial Policy Checklist.

Statistics

For all statistical analyses, confirm that the following items are present in the figure legend, table legend, main text, or Methods section.

n/a	Confirmed	
☒	☐	The exact sample size (*n*) for each experimental group/condition, given as a discrete number and unit of measurement
☒	☐	A statement on whether measurements were taken from distinct samples or whether the same sample was measured repeatedly
☒	☐	The statistical test(s) used AND whether they are one- or two-sided<br>*Only common tests should be described solely by name; describe more complex techniques in the Methods section.*
☒	☐	A description of all covariates tested
☒	☐	A description of any assumptions or corrections, such as tests of normality and adjustment for multiple comparisons
☒	☐	A full description of the statistical parameters including central tendency (e.g. means) or other basic estimates (e.g. regression coefficient) AND variation (e.g. standard deviation) or associated estimates of uncertainty (e.g. confidence intervals)
☒	☐	For null hypothesis testing, the test statistic (e.g. *F*, *t*, *r*) with confidence intervals, effect sizes, degrees of freedom and *P* value noted<br>*Give P values as exact values whenever suitable.*
☒	☐	For Bayesian analysis, information on the choice of priors and Markov chain Monte Carlo settings
☒	☐	For hierarchical and complex designs, identification of the appropriate level for tests and full reporting of outcomes
☒	☐	Estimates of effect sizes (e.g. Cohen's *d*, Pearson's *r*), indicating how they were calculated

Our web collection on statistics for biologists contains articles on many of the points above.

Software and code

Policy information about availability of computer code

Data collection	NA
Data analysis	Data was analyzed using publically avaialble methods, NA

For manuscripts utilizing custom algorithms or software that are central to the research but not yet described in published literature, software must be made available to editors and reviewers. We strongly encourage code deposition in a community repository (e.g. GitHub). See the Nature Portfolio guidelines for submitting code & software for further information.

Data

Policy information about availability of data

All manuscripts must include a data availability statement. This statement should provide the following information, where applicable:

- Accession codes, unique identifiers, or web links for publicly available datasets
- A description of any restrictions on data availability
- For clinical datasets or third party data, please ensure that the statement adheres to our policy

A data availability statement is included in the manuscript

Field-specific reporting

Please select the one below that is the best fit for your research. If you are not sure, read the appropriate sections before making your selection.

☐ Life sciences ☐ Behavioural & social sciences ☒ Ecological, evolutionary & environmental sciences

For a reference copy of the document with all sections, see nature.com/documents/nr-reporting-summary-flat.pdf

Ecological, evolutionary & environmental sciences study design

All studies must disclose on these points even when the disclosure is negative.

Study description	Identifying hydrocarbon degradation strategies across different redox conditions via metagenomic analysis of environmental and translationally active (BONCAT-FACS) samples from subsurface coal beds.
Research sample	Coal/groundwater slurry collected with a Subsurface Environmental Sampler from the Powder River Basin.
Sampling strategy	Samples were collected from one time point following either a nine or three month down-well incubation.
Data collection	HJS and HDS performed sample collection, sequencing and analysis.
Timing and spatial scale	Samples were collected at a single time point in 2017.
Data exclusions	NA
Reproducibility	All utilized methods are fully reproducible the only constraint is field site access which needs to be granted from the USGS.
Randomization	NA
Blinding	NA

Did the study involve field work? ☒ Yes ☐ No

Field work, collection and transport

Field conditions	Subsurface samples are stable and not impacted by surface weather fluctuations.
Location	Powder River Basin, Birney MT
Access & import/export	All samples were collected in the US and collection was performed with a USGS official on site.
Disturbance	Sampling were installed prior to this study and no disturbances resulted from the study.

Reporting for specific materials, systems and methods

We require information from authors about some types of materials, experimental systems and methods used in many studies. Here, indicate whether each material, system or method listed is relevant to your study. If you are not sure if a list item applies to your research, read the appropriate section before selecting a response.

Materials & experimental systems

n/a	Involved in the study	
☒	☐	Antibodies
☒	☐	Eukaryotic cell lines
☒	☐	Palaeontology and archaeology
☒	☐	Animals and other organisms
☒	☐	Human research participants
☒	☐	Clinical data
☒	☐	Dual use research of concern

Methods

n/a	Involved in the study	
☒	☐	ChIP-seq
☐	☒	Flow cytometry
☒	☐	MRI-based neuroimaging

Flow Cytometry

Plots

Confirm that:

☒ The axis labels state the marker and fluorochrome used (e.g. CD4-FITC).

☒ The axis scales are clearly visible. Include numbers along axes only for bottom left plot of group (a 'group' is an analysis of identical markers).

☒ All plots are contour plots with outliers or pseudocolor plots.

☒ A numerical value for number of cells or percentage (with statistics) is provided.

Methodology

Sample preparation	Following BONCAT incubation cells were removed from coal by removing 1 mL of slurry and adding 5mls Tween® 20 dissolved in PBS (1X PBS) to a final concentration of 0.02% (Sigma-Aldrich). Samples were vortexed at maximum speed for 5 min followed by centrifugation at 500 × g for 5 min (Couradeau et al., 2019) 38 . The supernatant (containing the detached cells) was immediately cryopreserved at −20 °C in sterile 55% glycerol TE (11X) solution. Translationally active cells were identified through a click reaction that added a fluorescent dye to HPG molecules that had been incorporated into newly synthesized protein. This BONCAT click reaction consisted of 5mM sodium ascorbate, 5mM aminoguanidine HCl, 500uM THPTA, 100uM CuSO4, and 5uM FAM picolyl azide in 1X phosphate buffered saline. Incubation time was 30 minutes, followed by three washes in 20ml of 1X PBS for 5 minutes each. Cells were recovered from the filter by vortexing in 0.02% Tween for 5 minutes, and then stained using 0.5uM SYTOTM59 (ThermoFisher Scientific, Invitrogen, Eugene OR, USA) DNA stain (Couradeau et al., 2019)
Instrument	BD-InfluxTM (BD Biosciences, San Jose, CA, USA)
Software	FlowJo (https://www.flowjo.com/) was used to analyzed saved sorting data
Cell population abundance	Sorted total and BONCAT+ samples demonstrated that 29.3 - 36.3% of the sorted cells belonged to populations that were BONCAT+ (as determined by comparison to controls)
Gating strategy	The instrument was configured to capture total cells labeled with SYTOTM59 DNA stain and excited with a 640nm red laser and BONCAT-positive cells labeled with FAM picolyl azide dye and excited with a 488nm blue laser. BONCAT-positive cells were a subset to total SYTOTM59 stained cells, and were differentiated from the BONCAT-negative based on their FAM fluorescence (530/40BP) to background fluorescence from identical cells inclubated without HPG, i.e., HPG negative control, that also underwent the same click reaction to add a FAM label.

☒ Tick this box to confirm that a figure exemplifying the gating strategy is provided in the Supplementary Information.
